# Poor adherence to iron-folic acid supplementation and associated factors among pregnant women who had at least four antenatal care in Ethiopia. A community-based cross-sectional study

**DOI:** 10.3389/fnut.2022.1023046

**Published:** 2022-12-08

**Authors:** Desale Bihonegn Asmamaw, Wubshet Debebe Negash, Desalegn Anmut Bitew, Tadele Biresaw Belachew

**Affiliations:** ^1^Department of Reproductive Health, Institute of Public Health, College of Medicine and Health Sciences, University of Gondar, Gondar, Ethiopia; ^2^Department of Health Systems and Policy, Institute of Public Health, College of Medicine and Health Sciences, University of Gondar, Gondar, Ethiopia

**Keywords:** adherence, associated factors, iron-folic acid, EDHS, Ethiopia

## Abstract

**Background:**

In developing countries, including Ethiopia, maternal mortality is a major public health concern. The Ethiopian Demographic Health Survey (EDHS) reported that the maternal mortality ratio (MMR) was 420 per 100,000 live births in 2016. Iron-folic acid supplementation (IFAS) is a key intervention to reduce these deaths. Therefore, this study aimed to assess the magnitude of poor adherence to IFAS and associated factors among pregnant women who had at least four antenatal care in Ethiopia.

**Methods:**

Secondary data analysis was used using 2016 Ethiopian Demographic and Health Survey (EDHS). We analyzed the data using Stata version 14. To identify factors associated with poor adherence to IFAS, a multilevel mixed-effect logistic regression model was fitted. Variables with a *p* < 0.05 in the multilevel mixed-effect logistic regression model were declared as significant factors associated with poor adherence to IFAS.

**Result:**

The magnitude of poor adherence to IFAS was 82.87% (95% CI: 80.96–84.65). Women education; primary [adjusted odds ratio (AOR) = 0.48; 95% CI: 0.31–0.75] and secondary (AOR = 0.52; 95% CI: 0.29–0.96), husband education; primary (AOR = 0.56; 95% CI: 0.36–0.86) and secondary (AOR = 0.51; 95% CI: 0.29–0.95), and community media exposure (AOR = 0.47; 95% CI: 0.27–0.79) were significantly associated with poor adherence to IFAS.

**Conclusion:**

In the current study, more than eight out of ten pregnant women who had at least four antenatal care had poor adherence to IFAS. Thus, promoting maternal and husband education and establishing community media with a priority on iron-folic acid supplementation and health-related programs are essential strategies to reduce poor adherence to IFAS.

## Introduction

The nutritional status of the mother during pregnancy has a significant impact on the health, development, and wellbeing of the child (1). Anemia is associated with maternal and infant morbidity and mortality during pregnancy since iron stores decrease and iron requirements increase (2). Anemia affects 38.2% of pregnant mothers worldwide, with Africa contributing 44.6%. Iron deficiency contributes to half of all anemia cases (3). Furthermore, more than one-fifth of maternal mortality in Sub-Saharan Africa (SSA) is indirectly attributable to anemia (4).

Iron deficiency is the major cause of anemia globally, particularly in SSA (5). Both the mother and the fetus can suffer from iron deficiency anemia during pregnancy (6). Preterm delivery, spontaneous abortion, low birth weight, and fetal distress are associated with anemia (6, 7).

Globally, anemia reduction is the second nutritional goal for 2025 and is a key component of achieving women's and children's health. The goal is to reduce anemia in women of reproductive age by 50% (8). Iron-folic acid supplementation (IFAS) is one of the most important interventions to reduce anemia among pregnant women (3, 8). A daily supplement containing 60 mg of elemental iron with 400 micrograms of folic acid is recommended for pregnant women by the World Health Organization (WHO) for 6 months (9). IFAS should be continued for three months postpartum in areas with high anemia prevalence (9). Furthermore, Ethiopia's national guideline for preventing and controlling micronutrient deficiency recommends taking IFAS daily for 6 months during pregnancy and 3 months after delivery (10). The Ethiopian national nutrition program (NNP II) also set a key target to increase the number of women receiving iron-folic acid supplements for more than 90 days during pregnancy to 50% by 2024 and 90% by 2029 (11, 12).

The poor adherence to IFAS varies across different settings, for instance, in urban and rural areas of Tigray, Ethiopia was 62.8 and 71.1%, respectively (13), Northeast, Ethiopia 52.4% (14), Shire refugee camps, Northern Ethiopia 35.3 (15), Tikur Anbessa Specialized Hospital, Ethiopia 36.4% (16), systematic review and meta-analysis in Ethiopia 58.6% (17), Tanzania 79.7% (18), and SSA 71.3% (19). Some of the identified factors that can affect poor adherence to IFAS include the age of the women, previous anemia, educational status, wealth index, knowledge about anemia, receiving counseling about nutrition, and husband support (13, 15, 17, 19–21).

The magnitude of anemia has persistently increased despite many efforts to fight it over the past three decades, including free provision of IFAS of mothers and the promotion of ANC. This can be supported by the evidence of EDHS, which found that the magnitude of anemia among reproductive-age women increased from 17% in 2011 to 24% in 2016. Only 5% of reproductive age women adhere to IFAS during their most recent pregnancy (22). Furthermore, various studies have been conducted in Ethiopia about IFAS (6, 7, 23–25). However, the majority of these focused on the effects of IFAS in newborns and pregnant women and only a limited number of studies assessed the adherence to IFAS and associated factors among pregnant women in Ethiopia. Nevertheless, there was no adequate literature that answered the reason why pregnant women who attended the recommended ANC had poor adherence to IFAS in the country. Therefore, this study aimed to determine the magnitude of poor IFAS and tried to explore factors determining adherence to IFAS among pregnant women who had at least four ANC.

## Methods

### Study design, setting, and sampling procedure

This study used data from the most recent Ethiopia Demographic and Health Surveys (EDHS). EDHS is a nationally representative household survey conducted every 5 years in low- and middle-income countries (26). Using the women's recode (IR file) data set, we extracted independent and dependent variables. The dataset is freely available for download at: https://dhsprogram.com/data/available-datasets.cfm.

**Figure 1:**
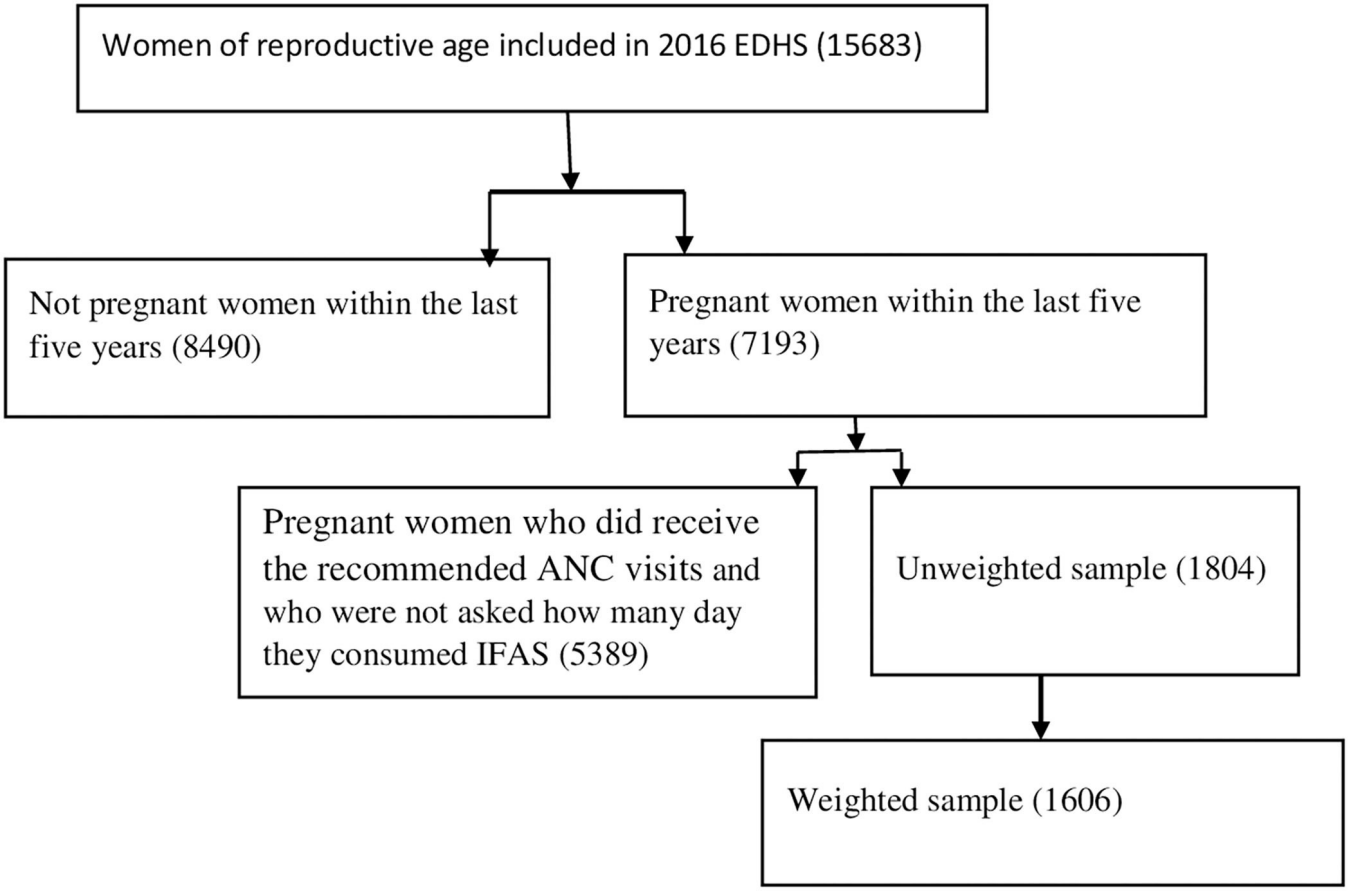
Data extraction procedures and sample size.

The EDHS employs two-stage stratified sampling technique. Which makes the data nationally representatives (7). A total weighted sample of 1,606 pregnant women within 5 years before the survey who had at least four antenatal care (ANC) were included in the study (Figure 1).

### Study variables

#### Outcome variable

The outcome variable for this study was poor adherence to iron-folic acid supplementation. It was defined as not using iron-folic acid supplementation for ≥90 days during the pregnancy of the most recent birth. This was measured in the DHS data by the number of days when iron supplements (tablets) were taken as part of antenatal care. The poor adherence of <90 days threshold was chosen in accordance with previous studies (19, 27, 28).

#### Independent variables

In this study, individual and community-level factors that are associated with poor adherence to IFAS were considered. Individual level factors considered in the analysis were age (15–24, 25–34, and 35–49), women education (no formal education, primary education, and secondary and above), husband education (no formal education, primary education, and secondary and above), occupation of the respondents (employed, non-employed), wealth index (poor, middle, and rich), nutrition counseling (yes, no), and religion (Orthodox, Muslim, protestant, and catholic). Regarding media exposure (yes, no), we coded yes if the women read newspaper, listened radio, or watched television for at least once a week, and no for otherwise (7).

Of the community level variables, region (small peripheral, large central, and metropolitan) and place of residences (rural, urban) were directly accessed from DHS data sets. However, community media exposure (low, high) and community-level education (low, high) were constructed by aggregating individual-level characteristics at the cluster level. They were categorized as high or low based on the distribution of the proportion values computed for each community after checking the distribution by using the histogram. The aggregate variable was not normally distributed, and the median value was used as a cut-off point for the categorization (29, 30).

### Statistical analysis

Data analysis was performed using Stata version 14. Before data analysis, all frequency distributions were weighted (v005/1000000) to ensure that the DHS sample was representative and to obtain accurate estimates and standard errors. In order to account for the hierarchical nature of the DHS data, a multi-level logistic regression analysis was conducted. First, bivariable multilevel logistic regression analysis was conducted and those variables with a *p* < 0.2 were considered for multivariable analysis.

After selecting variables for multivariable analysis, four models were fitted; null model (Model 0) which shows the variations in the poor adherence to IFAS in the absence of any independent variables. Model I adjusted for the individual-level variables, Model II adjusted for the community level variables and Model III adjusted for both individual and community level variables. Simultaneously, Model fitness was done using the deviance since these models were nested (20). In addition, Variance inflation factors (VIFs) were used to test for multicollinearity. Each independent variable had a VIF < 5, with a mean VIF of 1.84, indicating no significant multicollinearity. AORs were presented with a 95% confidence interval.

## Results

### Individual and community-level factors

A total weighted sample of 1,606 pregnant women were included in this analysis. The median age of the study participants was 28 years (IQR: 24–33) and 73.49% of the women were rural dwellers. Nearly half (48.31%) of the pregnant women had no formal education. Of the study participants, 49.19% were employed and 50.81 % had media exposure. The majority (88.07%) of the pregnant women were from large central regions. More than half (52.87%) of the pregnant women were from households with rich wealth quantiles (Table 1).

**Table 1:** Individual and community level factors associated with poor adherence to IFAS in Ethiopia.

**Variables**	**Weighted frequency**	**Percentage (%)**
**Women age**		
15–24	364	22.69
25–34	895	55.70
35–49	347	21.61
**Women education**		
No formal education	777	48.31
Primary education	517	32.21
Secondary education and above	313	19.49
**Women occupation**		
Employed	790	49.19
Unemployed	816	50.81
**Husband education**		
No formal education	538	36.06
Primary education	557	37.39
Secondary education and above	396	26.55
**Wealth index**		
Poor	481	29.96
Middle	276	17.17
Rich	849	52.87
**Media exposure**		
Yes	816	50.81
No	790	49.19
**Religion**		
Orthodox	873	54.37
Catholic	28	1.76
Muslim	309	19.25
Protestant	396	24.62
**Region**		
Small peripheral	57	3.56
Large central	1,415	88.07
Metropolitan	135	8.38
**Resident**		
Rural	1,180	73.49
Urban	426	26.51
**Community media exposure**		
Lower	983	61.2
Higher	623	38.80
**Community-women education**		
Lower	930	57.90
Higher	676	42.10

### Magnitude of poor adherence to IFAS

The magnitude of poor adherence to IFAS in Ethiopia was 82.87% (95% CI: 80.96–84.65).

### Factors associated with poor adherence to IFAS

The poor adherence to IFAS varied significantly across clusters. In the baseline model without an independent variable, 26.23 % of the variance in poor adherence to IFAS was explained by the variation in characteristics between clusters (ICC = 0.2623). In model 3, which included both individual and community level factors, the between-cluster variation, was reduced to 24.43%. Accordingly, the variance in IFAS adherence could be explained by differences in clusters. Model 3, which incorporated both individual and community-level factors, exhibited the best goodness of fit for predicting poor adherence to IFAS. The final model was selected because it has the lowest (1,250.90) deviance as compared with the other models (Table 2).

**Table 2:** Model comparison and random effect analysis result in Ethiopia.

**Parameters**	**Null model**	**Model I**	**Model II**	**Model III**
Variance	1.21	1.17	1.10	1.06
ICC (%)	26.23	25.06	26.16	24.43
PCV (%)	Ref	3.31	9.10	12.40
Log-likelihood	−696.30	−631.96	−687.75	−625.45
Deviance	1,392.60	1,263.92	1,375.50	1,250.90

In the multilevel multivariable logistic regression model, both the individual and community level factors were fitted simultaneously. Thus, women education, husband education, and community media exposure were statistically associated with poor adherence to IFAS at 95% confidence level.

This study showed that pregnant women attended primary and secondary education and above were 52% (AOR = 0.48; 95% CI: 0.31–0.75) and 48% (AOR = 0.52; 95% CI: 0.29–0.96) less likely to have poor adherence to IFAS compared to women with no formal education, respectively. Women with husband education attended primary and secondary education and above were 44% (AOR = 0.56; 95% CI: 0.36–0.86) and 49% (AOR = 0.51; 95% CI: 0.29–0.95) less likely to have poor adherence to IFAS compared to their counterparts, respectively. The odds of poor adherence to IFAS in the community with high media exposure were 53% (AOR = 0.47; 95% CI: 0.27–0.79) low compared to their counterparts (Table 3).

**Table 3:** Multivariable multilevel logistic regression model results of poor adherence to IFAS in Ethiopia.

**Variables**	**Null model**	**Model I (AOR, 95%CI)**	**Model II (AOR, 95%CI)**	**Model III (AOR, 95%CI)**
**Age of the respondent**				
15–24		1		1
25–34		1.28 (1.0.85, 1.92)		1.33 (0.87, 2.02)
35–49		0.90 (0.53, 1.52)		0.92 (0.55, 1.56)
**Women education**				
No formal education		1		**1**
Primary education		0.52 (0.33, 0.78)		0.48 (0.31, 0.75)^*^
Secondary education and above		0.53 (0.30, 0.93)		0.52 (0.29, 0.96)^*^
**Women occupation**				
Employed		0.91 (0.65, 1.28)		0.94 (0.66, 1.32)
Unemployed		1		1
**Husband education**				
No formal education		1		**1**
Primary education		0.57 (0.37, 0.87)		0.56 (0.36, 0.86)^*^
Secondary education and above		0.46 (0.27, 0.81)		0.51 (0.29, 0.95)^*^
**Wealth index**				
Poor		1		1
Middle		1.05 (0.63, 1.76)		1.06 (0.63, 1.77)
Riche		1.49 (0.94, 2.35)		1.67 (0.89, 2.05)
**Nutrition counseling**				
Yes		0.86 (0.57, 1.31)		0.88 (0.58, 1.33)
No		1		1
Religion				
Orthodox		1		1
Muslim		1.18 (0.73, 1.91)		1.26 (0.78, 2.07)
Protestant		1.14 (0.69, 1.92)		0.96 (0.56, 1.61)
Catholic		1.5 (0.26, 8.79)		1.32 (0.22, 7.70)
**Region**				
Small peripheral			1	1
Large central			1.25 (0.56, 2.81)	1.33 (0.56, 3.13)
Metropolitan			0.82 (0.31, 2.17)	0.83 (0.30, 2.29)
Resident				
Rural			0.81 (0.43, 1.51)	0.87 (0.45, 1.72)
Urban			1	1
**Community media exposure**				
Lower			1	**1**
Higher			0.44 (0.26, 0.73)	0.47 (0.27, 0.79)^*^
**Community-women education**				
Lower			0.98 (0.91, 1.07)	1
Higher			1.10 (0.68, 1.76)	1.63 (0.95, 2.72)

AOR, Adjusted Odds Ratio; PNC, Postnatal care; Model 1: adjusted for individual-level factors, Model 2: adjusted for community-level factors, Model 3: adjusted for both individual and community-level factors (full model), and

* Statistically significant at p < 0.05 in the full model.

Bold value indicate statistical significant.

## Discussion

The aim of this study was to determine poor adherence to IFAS and identify associated factors among pregnant women who had at least four ANC. This study found that 82.87% (95% CI: 80.96–84.65) of pregnant women who had at least four ANC had poor adherence to IFAS. The result of the study found that women's education, husband's education, and community-level media exposure were identified as the factors associated with poor adherence to IFAS.

The current study is lower than a study conducted in Ethiopia 87.6% (7). The possible explanation might be the difference in the study population. The current study exclusively included pregnant women who received the recommended ANC, whereas the previous study included pregnant women who were asked how many days they consumed IFA tablets regardless of the number of ANC visits (7). Women who received the recommended ANC visits had better knowledge about anemia compared to those who did not receive the recommended ANC visits (31). Previous studies have documented that ANC visits and knowledge of anemia have a negative relationship with poor adherence to IFAS (7, 23–25, 32). However, this finding is higher than that of studies conducted in Ethiopia (23–25, 33–35), Tanzania 79.7% (18), and sub-Saharan African countries 71.3% (19). The possible explanation could be because of the difference in the study setting, the quality of service delivery in the health institutions, socio-demographic differences, and women's awareness of the importance of IFAS during pregnancy. For example, the previous studies done in Ethiopia were small-scale surveys compared to the EDHS survey, which was a national representative survey and included developing regions. Regarding socio-demographic variation, a previous study done in SSA reported that only 34.4% of the pregnant women had no formal education, which was lower than that of the current study (48.3%). Previous research has shown that women's education has a negative relationship with poor adherence to IFAS (21, 24, 36). Furthermore, the reason could also be the difference in access to health institutions and the availability of IFA in the nearby health facilities (7).

Pregnant women with primary education and secondary education and above were decreases poor adherence to IFAS by 52 and 48% as compared to those with no formal education, respectively. This result is in line with previous studies done in Ethiopia (24, 36), and Indonesia (37). Educated women are better informed about iron deficiency anemia and therapy, supplement benefits, and pregnancy in general. In addition, education may enhance awareness of micronutrient deficiency and ways to overcome it (7, 38).

Pregnant women with husband education who had formal education were less likely to have poor adherence to iron supplementation than those pregnant women with husbands who had no formal education. The finding is consistent with other study done in Ethiopia (39). This might be due to mothers with husband who were educated were autonomous on utilization of iron supplementation without the consent of their husbands.

Community media exposure is also negatively affected with poor adherence to IFAS. This study was supported by studies conducted in Ethiopia (40), and Asia (41). This is because different maternal and child health services including the importance of iron-folic acid supplementation during pregnancy are frequently given to the community through mass media. Therefore, pregnant women who are exposed to community media would have better understanding of the advantages of IFAS during pregnancy compared to those who do not have community media exposure (40).

## Strengths and limitations

This study used nationally representative data sets, which were collected with a standardized and validated data collection tools. A multilevel analysis was used in this study to account for the hierarchical nature of the data. The cross-sectional nature of the survey does not show the causal relationship between outcome and independent variables. Furthermore, due to the use of secondary data, essential factors like knowledge of anemia, and socio-cultural factors were not available in the EDHS data set, hence, they were not included in the analyzed.

## Conclusion

In the current study, more than eight out of ten pregnant women who had at least four antenatal care had poor adherence to iron-folic supplementation. Thus, promoting maternal and husband education and establishing community media with a priority on iron-folic acid supplementation and health-related programs are essential strategies to reduce poor adherence to iron-folic acid supplementation.

## Data availability statement

The raw data supporting the conclusions of this article will be made available by the authors, without undue reservation.

## Ethics statement

Ethical review and approval was not required for the study on human participants in accordance with the local legislation and institutional requirements.

## Author contributions

DA conceived the idea, extract the data, data analysis, and draft the manuscript. WD, TB, and DB participant in the data analysis, interpretation, and revising of the manuscript. The final manuscript has been read and approved by all authors.
